# Antimicrobial peptide similarity and classification through rough set theory using physicochemical boundaries

**DOI:** 10.1186/s12859-018-2514-6

**Published:** 2018-12-06

**Authors:** Kyle Boone, Kyle Camarda, Paulette Spencer, Candan Tamerler

**Affiliations:** 1 Bioengineering Program, Institute of Bioengineering Research, University of Kansas, Learned Hall, Room 5109, 1530 W 15th Street, Lawrence, KS 66045 USA; 2 Chemical and Petroleum Engineering Department, University of Kansas, Learned Hall, Room 4154, 1530 West 15th Street, Lawrence, KS 66045 USA; 3 Mechanical Engineering Department, Bioengineering Program, Institute of Bioengineering Research, University of Kansas, Learned Hall, Room 3111, 1530 West 15th Street, Lawrence, KS 66045 USA; 4 Mechanical Engineering Department, Bioengineering Program, Institute of Bioengineering Research, University of Kansas, Learned Hall, Room 3135A, 1530 W 15th St, Lawrence, KS 66045 USA

**Keywords:** Antibacterial peptides, Classification, Machine learning, Physicochemical properties, Rough set theory, Sequence similarity, Supervised learning, Functional peptide search

## Abstract

**Background:**

Antimicrobial peptides attract considerable interest as novel agents to combat infections. Their long-time potency across bacteria, viruses and fungi as part of diverse innate immune systems offers a solution to overcome the rising concerns from antibiotic resistance. With the rapid increase of antimicrobial peptides reported in the databases, peptide selection becomes a challenge. We propose similarity analyses to describe key properties that distinguish between active and non-active peptide sequences building upon the physicochemical properties of antimicrobial peptides. We used an iterative supervised machine learning approach to classify active peptides from inactive peptides with low false discovery rates in a relatively short computational search time.

**Results:**

By generating explicit boundaries, our method defines new categories of active and inactive peptides based on their physicochemical properties. Consequently, it describes physicochemical characteristics of similarity among active peptides and the physicochemical boundaries between active and inactive peptides in a single process. To build the similarity boundaries, we used the rough set theory approach; to our knowledge, this is the first time that this approach has been used to classify peptides. The modified rough set theory method limits the number of values describing a boundary to a user-defined limit. Our method is optimized for specificity over selectivity. Noting that false positives increase activity assays while false negatives only increase computational search time, our method provided a low false discovery rate. Published datasets were used to compare our rough set theory method to other published classification methods and based on this comparison, we achieved high selectivity and comparable sensitivity to currently available methods.

**Conclusions:**

We developed rule sets that define physicochemical boundaries which allow us to directly classify the active sequences from inactive peptides. Existing classification methods are either sequence-order insensitive or length-dependent, whereas our method generates the rule sets that combine order-sensitive descriptors with length-independent descriptors. The method provides comparable or improved performance to currently available methods. Discovering the boundaries of physicochemical properties may lead to a new understanding of peptide similarity.

**Electronic supplementary material:**

The online version of this article (10.1186/s12859-018-2514-6) contains supplementary material, which is available to authorized users.

## Background

In the US, over 23,000 deaths each year are associated with drug-resistant bacterial infections [1]. These types of infections are central to the projected increase in deaths globally by 2050, which are expect to reach 10 million annually [2, 3]. The rise of antibiotic-resistant bacteria has prompted increasing interest in antimicrobial peptides as a solution to this critical issue [4]. Over 2800 antimicrobial peptides have been discovered from natural sources in the last decade [5–11]. Antibacterial peptides derived from these natural sequences have shown both broad-spectrum and improved activity against targeted bacteria [12–16]. Antibacterial peptide-mimics are introduced as another source to the existing peptide libraries by incorporating additional backbone chain atoms for more structural flexibility and resistance to protease degradation [17–20]. This list extends by exploring the post-translationally modified antimicrobial peptides offering chemical properties beyond the naturally occurring amino acids [21, 22].

While many antimicrobial peptides have been discovered at the laboratory bench, computational methods have been integrated into this search to find many more candidates. Encrypted antimicrobial peptides are an example in which known active peptides are queried against DNA repositories to find new antimicrobial peptides [23]. Among many methods, grammar-based methods and regular-expression-based match sequence patterns are used to identify functional similarity [24, 25]. Computer-aided molecular design [26–29] approaches using quantitative sequence activity relationships [30–33] (QSAR) predict the antibacterial level of peptides given key chemical properties. Artificial neural networks (ANN) have been used both to generate new sequences and to distinguish between active and inactive sequences [25, 34–37]. They are often used in the classification of antimicrobial peptide sequences [7, 38]. While ANNs are flexible enough to model many kinds of complex relationships, they lack transparency about how classification choices are made. Determining the boundaries of the similar antimicrobial peptide clusters remains difficult despite many existing machine learning methods.

Due to the ongoing need for improved antimicrobial peptide selection and design, many classification approaches have been developed with supervised machine learning methods. A recent review by Porto et al contrasts two different kinds of sequence representations for antibacterial classification [25]. The first kind of representation preserves the order of the sequence which tends to lead to length-dependent predictions [39]. False positives may be produced if the overall chemical properties of an antibacterial peptides are changed by adding amino acids with contradictory chemical properties. The second kind of sequence representation preserves overall sequence properties which tends to lead to order-insensitivity. False positives may be produced if the order of an active peptide is scrambled [24].

AntiBP [40] was one of the first online available services for antibacterial peptide prediction. AntiBP uses a sliding window of 15 residues to predict the classification using support vector machines (SVM) [41], quantitative matrices (QM) [42] and artificial neural networks (ANN) [43]. The strength of this approach is that the order of amino acids impacts the prediction. However, the weakness to having a constant window of amino acids is that the predictions are peptide-length dependent [39]. To overcome the peptide length dependence, another method CAMP (Collection of Antimicrobial Peptides) [44] was employed to use descriptors that summarize composition, physicochemical properties and structural features of the peptides. CAMP uses multiple machine learning approaches for these features such as SVM [45], ANN [46, 47], discriminate analysis (DA) [48] and random forest (RF) [49]. However, the descriptor approach is insensitive to the sequence order arrangement. For example, full-length sequence descriptors can be sensitive to the overall charge of a peptide but not its charge distribution. iAMP-2 L (antimicrobial peptide prediction two-level) [50] partially addresses the order insensitivity by calculating the autocorrelation of amino acid property values within the amino acid sequence. Other descriptors do not account for the order of the sequence [24]. Because the iAMP-2 L classification algorithm is based on a fuzzy *K*-nearest neighbor algorithm, clusters that are invariant for descriptors that include correlations would be sequence-order insensitive. This approach is also sequence-order insensitive to sequence rearrangements that preserve the correlation structure from the original peptide. Evolutionary Feature Construction [51–53] (EFC) method addresses this need by achieving order-sensitive classification by combining order sensitivity and length independence by selecting common chemical property sequence patterns for antimicrobial peptides. Length-independent classification is achieved with a support-vector machine method through physicochemical descriptors selected by FCBF (Fast-Correlation Based Filter selection) [52]. While this method does combine order-sensitivity and length-independence, it does not completely address either of these issues. Order-insensitivity is possible based on the rearrangements of amino acids that are indistinguishable by the pattern recognition scheme of compressing 20-amino acids into four categories.

We propose a novel method that addresses order sensitivity by calculating the physicochemical properties of sub-sequences in addition to using descriptors of physicochemical properties for length independence. Our method therefore combines order-sensitivity and length independence as a new approach. We analyze these descriptors using rough set theory (RST). Rough set theory is a heuristic method for discovering rules, which distinguish between outcomes. These rules show which data features and data values are useful to distinguish between outcomes. To the best of our knowledge, RST has not yet been studied to classify peptide or protein sequences based on their activity. Our RST implementation uses features that summarize the physicochemical properties of the full-length sequences, which are sequence-order insensitive, and features which summarize constant-length subsequences, which are sequence-order sensitive. RST selects combinations of both kinds of descriptors into a single rule. Each rule defines its own cluster including the classification of the peptide’s activity or inactivity.

Using a rough set theory approach that combines the algorithm of MLEM2 (modified learning from examples module, Version 2) [54] with the algorithm IRIM (Interesting Rule Induction Module) [55], we developed a method that investigates the sequence-function relationships. The main difference in from other RST methods is that it uses local coverings to generate rules, which are different from the lower and upper approximations in the basic RST methodology. IRIM is a method that optimizes for rules that have the most training set sequences that apply. This is different from MLEM2 in that IRIM may not provide a rule that applies to every training set sequence. We achieve high specificity performance with our condition-limit number MLEM2 with the fewest chemical property features among benchmarked methods. Our method was tested against publicly available prediction servers CAMP AMP prediction [9], iAMP-2 L [50], and a motif-searching algorithm EFC method [51, 52] with and without FCBF. The approach produces physicochemical boundaries that create definitions of similarity among antimicrobial and non-antimicrobial peptides.

## Results

The explosion of available antimicrobial peptides brings the new challenge of selecting which antimicrobial peptides to use [38, 56–58]. With the large increase in the number of available peptides, there is an opportunity to classify peptides with respect to their similarity. We define similarity by the physicochemical properties of the peptides, which we show can differentiate between active and inactive peptides. Each rule generated is a category of peptides with boundaries of physicochemical properties chosen so that no rule category is a mixture of active and inactive peptides beyond an allowed limit. We generate rules until all peptides in the training set are covered by at least one category.

**Fig. 1:**
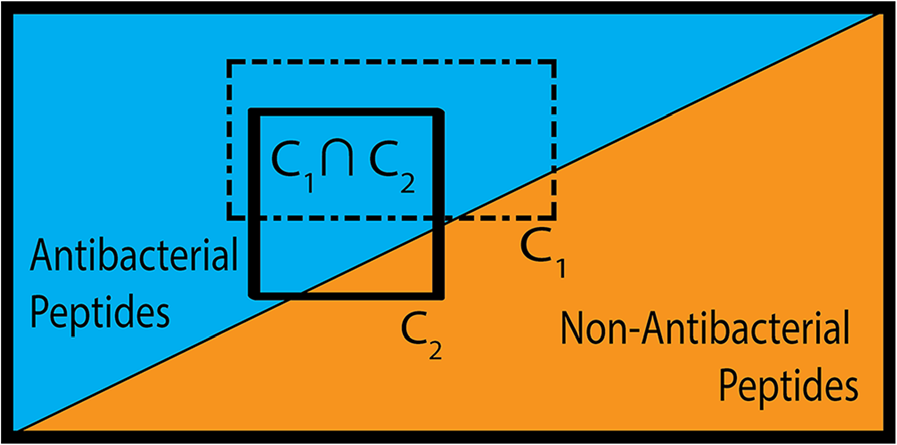
Rough Set Theory Rule Generation. A) Venn diagram of active and inactive peptides. A rule (R_1_) is the intersection of conditions (C_1_and C_2_). Each rule must be selective for either active or inactive peptides. The minimum accuracy allowed for a rule is a user-defined parameter α. B) Venn diagram showing multiple rules as the intersection of conditions in 2-D space. The selection of conditions that lead to rules is a feature selection process that chooses the most relevant conditions to describe the physicochemical boundaries. A rule set is the collection of all rules describing the boundaries for either activity or inactivity

Training sets are formatted as data tables; Table 1 is provided as an example to summarize these data sets. The first column is the identity column, which presents the sequences of the peptide. Each row of the data table corresponds to one peptide sequence. The feature columns list the corresponding values for each peptide depending on the amino acid properties and the summarizing function. The final column is the label of antibacterial activity. A condition is a value interval for a feature. The intersection of conditions is a rule, as shown in Fig. 1.

**Table 1:** Schematic Data table representing the training data set before feature correlation analysis. The three sections of the table are the sequences from iAMP-2 L training set [50], the features derived from the 544 amino acid properties in the AAindex1 [63], and the classification label of antibacterial activity from the positive or negative training data set. a_n_ denotes a sequence, b_n_ indicates the sum of the sequence for an AAindex1 property, c_n_ indicates the mean and d_n_ indicates the maximum sum of three adjacent residues in the sequence

Sequence	Sum of	Mean of	Window of	Antibacterial Activity
	A_1_ …A_544_	A_1_ …A_544_	A_1_ …A_544_	
a_1_	(b_1_)_1_…(b_1_)_544_	(c_1_)_1_…(c_1_)_544_	(d_1_)_1_…(d_1_)_544_	Active
…	…	…	…	Active
a_1274_	(b_1274_)_1_…(b_1274_)_544_	(c_1274_)_1_…(c_1274_)_544_	(d_1274_)_1_…(d_1274_)_544_	Active
a_1,275_	(b_1,275_)_1_…(b_1,275_)_544_	(c_1,275_)_1_…(c_1,275_)_544_	(d_1,275_)_1_…(d_1,275_)_544_	Inactive
…	…	…	…	Inactive
a _2714_	(b _2714_)_1_…(b _2714_)_544_	(c _2714_)_1_…(c _2714_)_544_	(d _2714_)_1_…(d _2714_)_544_	Inactive

Evaluating the performance of the rules being generated is performed by calculating the *Pr*, the training set accuracy performance of the rule. The *Pr* is the ratio of the size of the sets of peptides described by the intersection of all the conditions in the rule that meet the targeted label to all the peptides described by the intersection of the conditions (Eq. 1). The CLN value is the user-defined condition-limit number, which limits the number of conditions in each of the rules. The value of *Pr* must be at or above α, the user-defined minimum training accuracy a rule must have to be included in the rule set.

1 $$ \mathit{\Pr}=\frac{{\left|{\bigcap}_1^{CLN}{C}_i\right|}_{targeted\ label}}{{\left|{\bigcap}_1^{CLN}{C}_i\right|}_{any\  label}} $$

In using the rough set theory approach, we modified existing approaches by combining the features of MLEM2 (modified learning from examples module, Version 2) method [59, 60] with a feature of the module IRIM (Interesting Rule Induction Module) to potentially improve our selectivity and specificity [61]. We modified the MLEM2 method by adding the ability to limit the condition number for each of the rules, a feature of IRIM. Because the IRIM method exhaustively searches all possible rules given the number of conditions, it cannot be used for large numbers of conditions or large numbers of peptides because the runtime grows exponentially with the number of conditions.

Our modified MLEM2 method uses the heuristics of the MLEM2 method to select condition combinations with a run time that grows polynomially in the number of peptides and in the number of conditions. Our modified method includes a defined-condition number (CLN) which combines the polynomially-bound worst-case runtime of MLEM2 with the set number of conditions of IRIM. Because a small number of conditions are selected from the available number of conditions, CLN-MLEM2 is an embedded feature selection method [62]. It attempts to use the most relevant conditions to describe the boundaries. The relevance of a condition is the number of peptides that are described by it in the training set. The CLN-MLEM2 method selects rules based on a user-defined minimum accuracy referred to as α (0 ≤ α ≤ 1). Using higher values of α generates fewer rules with higher *Pr* values of training accuracy. Using lower values of alpha generates more rules with lower *Pr* values of training accuracy. CLN-MLEM2 generates rules until all peptides in the training set have at least one rule that applies to it. The collection of all rules for either active peptides or inactive peptides is called a rule set.

**Fig. 2:**
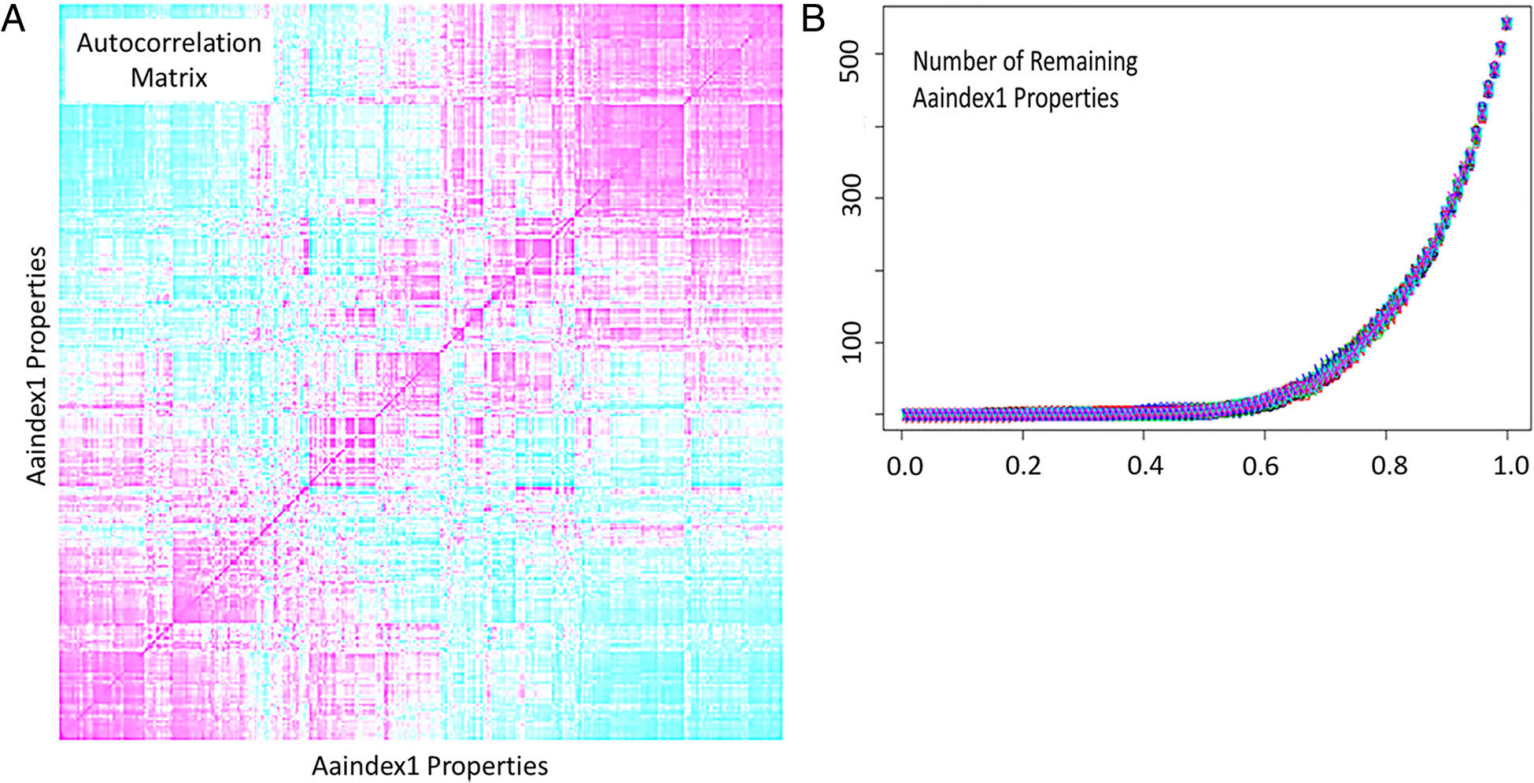
Auto-Correlation and Selection of AAindex1 Properties. **a** Auto-correlation plot of 544 different AAindex1 properties. Magenta represents positive correlation, cyan represents negative correlation and white represents the lack of correlation between properties. **b** Remaining number of AAindex1 properties following filtering by cut-off value for the absolute value of correlation

To begin the defined-condition number MLEM2 (Modified Learning from Experience Module 2) method, we generate multiple summaries of the amino acid sequences of the given active and inactive peptides by selecting non-correlated amino acid properties in the AAindex1 [63] (Amino Acid index 1). Among the 544 properties of the AAindex1, many of the properties are highly correlated. The autocorrelation matrix of the AAindex1 properties was calculated as the pairwise Pearson correlation value of each pair of properties in the index. The heat map of correlation values for the autocorrelation matrix is shown in Fig. 2a. Positive correlation is magenta and negative correlation is teal. Non-correlated amino acid property pairs are white. The autocorrelation matrix shows that most amino acid properties are highly correlated. We studied how many amino acid properties are below a correlation threshold for all other amino acid properties (Fig. 2b). We performed 60 repetitions with random initial properties of eliminating properties more correlated than a threshold. We found a very tight trend of how many uncorrelated properties there are for a given cut-off value. For further study, we selected a correlation cut-off of 0.65, which resulted in 74 properties remaining from the original 544 properties.

We seek to combine overall sequence chemical properties and motif properties to be able to account for how all of the residues may affect the chemical properties while still retaining the ability to separate classifications based on the ordering of the residues. If only chemical properties are evaluated by the sum or mean of the whole sequence, then the rules generated are sequence-order insensitive. By considering sub-sequences of the peptides, then the ordering of the chemical properties within the sequence can be used as a feature. We calculate two types of sequence property summaries from the selected amino acid properties in the AAindex1 (Amino Acid index 1) after removing the correlated amino acid chemical properties. First, we calculate overall property summaries as the mean and average of the properties of the amino acids present in the sequence. Secondly, we calculate motif properties as the maximal subsequence sum of a given length of the amino acid sequence. Our CLN-MLEM2 method can combine overall sequence properties and motif properties within a single rule. Each rule forms a class of either active or inactive peptides.

We used previously studied, publicly available datasets of antimicrobial peptides [50, 64] to test our method of finding physicochemical boundaries for antibacterial activity. See Table 2 for the inducted rule category with the largest membership of the studied dataset. The rule category is the conjunctive expression of each of the conditions up to the user-defined condition-limit number (CLN) with the rule applying to antimicrobial peptides whose property values are within the range of the values given in Table 2 (Eq. 2). This rule has a high selectivity of 97.8% with a false discovery rate of 2.2%. All sequences that do not match any rule for the applied rule set are classified as non-antibacterial.

2 $$ \bigcap \limits_1^{n= CLN}\left({Lower\ Value}_{condition}\le {Value}_{peptide}\le {Upper\ Value}_{condition}\right)\overset{predicts}{\to } Antibacterial\ Activity $$

**Table 2:** Rough set theory rules generated with maximum support from large training dataset. The first rule describes antibacterial sequences. The accuracy of this rule is 97.8% (446/456) for the peptides that met the conditions from either the dataset from Xiao, et al [50] or the dataset from Fernandes, et al. [64]

Calculation	AAindex1 Property	Lower Value	Upper Value
Window 3	NAKH900111	31.21	48.66
Window 3	FINA910104	3.45	5.10
Window 3	KUMS000101	23.6	28.20
Sum	GEIM800102	12.68	39.90
Window 3	VASM830102	1.67	2.12
Window 3	QIAN880139	0.38	0.98
Sum	FAUJ880112	0	3
Sum	CHAM820102	−0.61	19.51

## Discussion

Protein and peptide sequence-based classification methods have been extensively developed to improve the understanding of the functionality of polypeptides [65, 66]. By using rough set theory, our method builds rules that distinguish between active antibacterial peptides from inactive antibacterial peptides. The developed method was benchmarked against methods including a recently published method EFC [52], based on motif-recognition, as well as against a larger set of methods from publicly available prediction servers. The first benchmark test is a ten-fold cross validation on a dataset used in previous studies [52, 64] with the positive sequences clustered from the APD2 (Antimicrobial Peptide Database 2) [10] to 115 clusters and the negative sequences from the PDB [67] clustered to 116 clusters. Each cluster is represented by one sequence. The results were compared with EFC-based methods and support vector machines given subsequences of lengths 5 to 8 amino acids. Table 3 demonstrates that our method has high selectivity and accuracy in comparison to the performance of the SVM methods, and comparable selectivity and accuracy in comparison to the EFC method. A trend of decreasing Mathew’s Correlation Coefficient (0 for random guessing and 1 for perfect performance) as the length of the subsequence increases is seen in Table 3. Our subsequences in CLN-MLEM2 are 3 amino acids long and may have helped to contribute to our improved performance for using a single length of subsequences instead of combining four different lengths in the EFC method.

**Table 3:** Performance of rough set theory rule induction compared to motif-search in 10-fold cross validation

Method	Sensitivity (%)	Specificity (%)	MCC
5-kmer SVM	75.7	75.0	0.54
6-kmer SVM	74.8	74.1	0.46
7-kmer SVM	73.0	72.4	0.40
8-kmer SVM	73.0	72.4	0.36
EFC-FCBF	87.1	87.2	0.76
CLN-MLEM2	86.9	86.3	0.75

We further tested our modified MLEM2 method against a larger variety of classification methods. The second benchmarking test uses the iAMP-2 L dataset [50]. Like the dataset used for the first benchmark, this dataset is derived from the APD2 database. However, instead of choosing a single sequence from each cluster, the sequences were narrowed by removing sequences with greater than 40% similarity as measured by CD-HIT [68] only with cluster of more than 250 sequences. This resulted in a testing positive dataset of 848 unique sequences. The negative sequences were from a UniProt search of cytoplasmic proteins, also with less than 40% similarity. 2405 unique sequences were included in the negative dataset. The positive training data set was the S1 set (“Antibacterial”) from iAMP-2 L, which has 1274 unique sequences. The negative training set of data was the non-AMP data set from iAMP-2 L, which has 1440 unique sequences.

While our method has comparable selectivity in classification to current state-of-the-art method, our method is among the best in specificity (Table 4). The combination evolutionary algorithm with chemical properties (EFC + 307-FCBF: EFC combined with FCBF (Fast Correlation Based Features) using 307 features) is the only other state-of-the-art method with specificity that is comparable to ours. We achieve similar specificity using 74 AAindex1 features instead of 307 AAindex1 features. Removing the length-independent representation from the EFC method (EFC-FCBF: EFC without FCBF) results in almost no loss of sensitivity, but a loss of 6% in selectivity. Removing the order-sensitive representation for EFC in Table 2 results in lower sensitivity and selectivity performance (MCC = 0.54). While the datasets are different, between Table 3 and Table 4 results, the difference in the individual components of the EFC algorithm compared to the combined algorithm shows a dramatic improvement when integrating order-sensitive and length independent sequence representations. Our CLN-MLEM2 method integrates these two types of representations at its most basic level of output, the rule.

**Table 4:** Performance comparison among prediction servers for antimicrobial peptides, a motif-based classification method and rough set theory approach

Method	Sensitivity (%)	Specificity (%)	MCC
CAMP SVM	95.8	39.8	0.43
CAMP RF	97.1	33.5	0.40
CAMP ANN	89.1	70.9	0.61
CAMP DA	94.1	49.5	0.49
iAMP-2 L	97.7	92.0	0.90
EFC-FCBF	92.0	90.0	0.73
EFC + 307-FCBF (307 AAindex1 features)	92.4	96.1	0.86
CLN-MLEM2 (74 AAindex1 features)	88.0	95.4	0.85

**Fig. 3:**
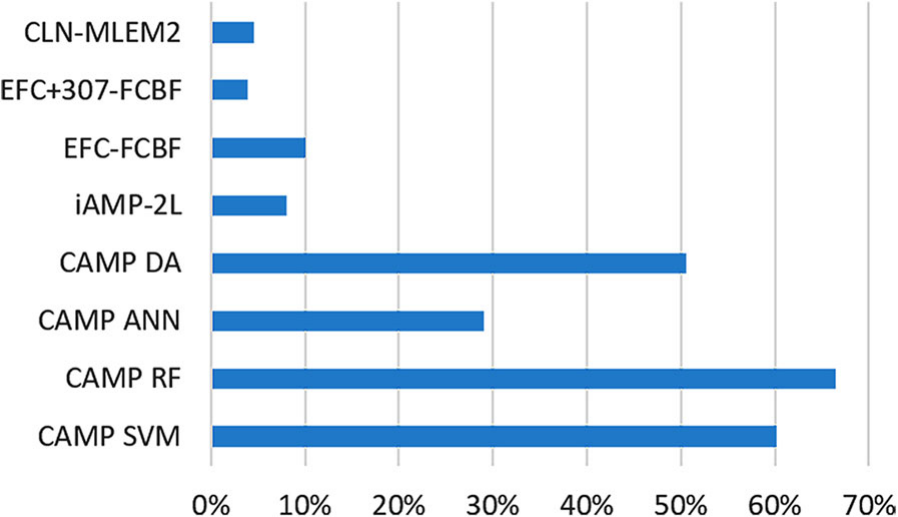
False discovery rates of comparative antimicrobial peptide classification methods. CLN-MLEM2 achieves a low false discovery rate among currently available antimicrobial peptide classification methods

Our method has high specificity and similar accuracy for antibacterial classification as other current methods. When using a classification method for the discovery of antimicrobial peptides, the specificity of the method is more important than its selectivity [69]. Our method prioritizes specificity with low false discovery rate (FDR) by classifying sequences that do not meet any rule in the applied rule set as inactive (Fig. 3). In fact, there is only one method, which provides lower FDR compared to our method, i.e. EFC + 307-FCBF. However, our method results in similar specificity starting with fewer physicochemical properties. The robustness of this method may be potentially improved with ensemble learning and voting scheme approaches. If our method provides unique descriptions of activity, then it will reduce the overall false discovery rate of the ensemble method and voting scheme approaches.

CLN-MLEM2 has been shown to be useful for the learning task of predicting antibacterial activity from a peptide sequence. This learning task is related to multi-instance learning. A classic literature example of a multi-instance learning problem is in drug activity prediction [70]. Active molecules have at least one conformation that interacts with a drug target, while inactive molecules have none. The challenge is to identify which conformations interact with the drug target. Each drug has one molecular formula, but it can have many conformations. Each peptide also has one sequence but many physicochemical property values. The CLN-MLEM2 method has found the most relevant physicochemical property features that relate to the activity of the peptide sequence. This CLN-MLEM2 method can also be applied to the multi-instance learning case of describing the conformations of peptides are active.

Our method also acts as an embedded feature selection tool by limiting the physicochemical properties in the rules to a user-defined number [62]. This embedded feature selection property may make CLN-MLEM2 useful for feature selection for other methods in the field, with the capability of setting the limit of the number of features to select. Our proposed method, CLN-MLEM2 has a low false discovery rate compared to comparative antimicrobial peptide methods as shown in Fig. 3. EFC method also has a low false discovery rate when including the physicochemical properties, but a doubled false discovery rate when the pattern recognition component is used alone.

A decrease in selectivity of the classification will cause longer computer search times, while a decrease in specificity will increase the number of necessary experimental activity assays. Since the cost of experimentally testing peptides is much greater than the computational time of searching for antimicrobial peptides, methods that have high specificity are preferred. In addition to the high specificity of our method, our method creates categories of antimicrobial peptides. Categorization of peptides aids in the selection and in the design of antimicrobial peptides by providing similarity groupings according to physicochemical property boundaries. Peptides that match multiple active categories can combine more physicochemical property values associated with activity.

## Conclusion

The increase in multidrug resistant bacteria usage has prompted an intense search for agents that can be used to treat infectious diseases. There is growing interest in antimicrobial peptides as novel agents to treat infections, and this interest has led to an exponential growth of known antimicrobial peptides. However, peptide selection is becoming another challenge with the drastic increase in the number of these peptides discovered from natural resources, their modified version as well as computational derived ones. We developed a method, CLN-MLEM2, for generating rule sets to describe the similarity among antimicrobial peptides by physicochemical boundaries. Our CLN-MLEM2 method allows the user to limit the number of physicochemical properties used to set the boundaries. Discovering where the boundaries of physicochemical properties are among active peptides generates new categories of antimicrobial peptides.

Our approach simultaneously groups peptides and classifies them. We benchmark our rule set performance to other classification methods. Some available classification methods are either sequence-order insensitive or length-dependent. The rule sets our method generates combine order-sensitive descriptors with length-independent descriptors. We achieve comparable or improved specificity and selectivity to currently available methods with lower false discovery rates. The high specificity of our method aids novel antibacterial peptide discovery because a low false discovery rate reduces the number of bacterial assays.

## Methods

In this study we test our rough set theory classification method to differentiate antibacterial peptides from APD2 [10] (Antimicrobial Peptide Database 2) and randomly selected peptides from the UniProt database [71, 72]. These benchmark datasets are available online [50, 64].

### Rule induction by the MLEM2 algorithm

The MLEM2 rule induction method [54] is a classification method based on a rough set theory approach that uses local approximations of concepts to generate rules when the available attributes cannot perfectly separate the data. A local approximation is finding collections of conditions that cover a concept with an accuracy requirement parameter α. We use a modified MLEM2 version that combines the polynomial run time growth rate of MLEM2 with the defined-condition number of the IRIM (Interesting Rule Induction Method) to find rules with small numbers of conditions in large datasets with many attributes. IRIM has an exponential run time growth rate with respect to attribute number. We set the maximum number of conditions to be eight (8). Conditions are intervals of feature values. Each peptide sequence has one value for each feature. Rules are conjunctive expressions of conditions.

**Fig. 4:**
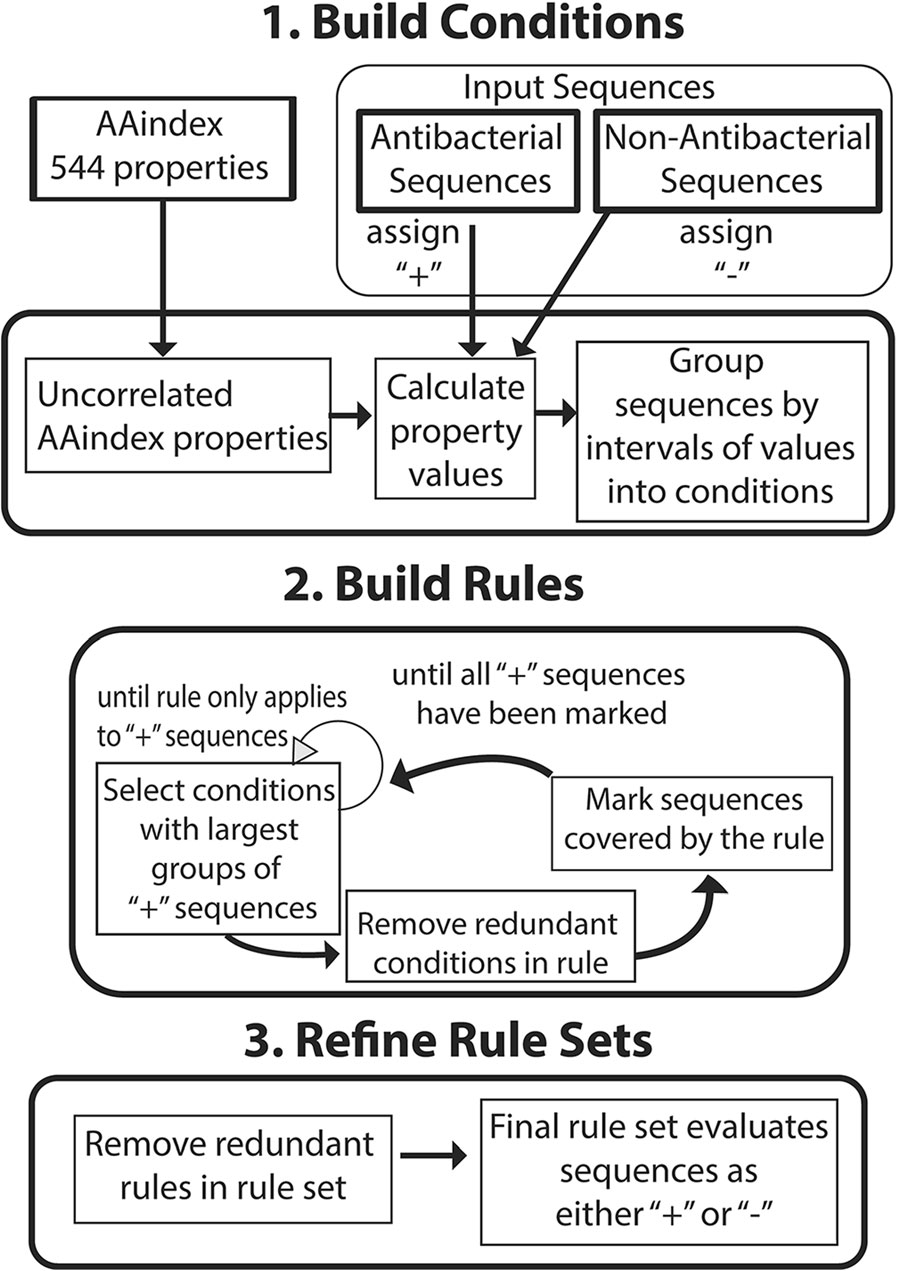
CLN-MLEM2 Method. CLN-MLEM2 Rule induction process based on rough set theory approach to classify peptides with antibacterial activity

Figure 4 shows the overall process for building rules. Rules are built from conditions that contain the maximum number of peptide sequence of the desired antibacterial label. Ties are broken by the conditions that have the highest percentage of peptide sequences with the desired antibacterial label. Rules are refined by narrowing the interval of an included condition or by adding a new condition to the conjunctive expression. Rules are simplified by omitting redundant conditions whose loss still results in a rule with no loss of accuracy. The minimum accuracy that a valid rule must have is a user-defined value, α. In this study, α is set to the accuracy of the majority class rule, which is to label all peptides with the non-antibacterial class.

Table 5 shows a compact data table that is consisted of six sequences with two features to illustrate methodology. The most relevant condition among the two features for active antibacterial activity is the sum of the positive charge from 1 to 3, relating to all three active peptides. This condition does not form a rule, however there is an inactive sequence with a sum of positive charge of 1. To distinguish between inactive and active between these two sequences, the second feature of the sum of negative charges is considered. The intersection of the conditions of the sum of positive charge from 1 to 3 and the sum of negative charge from 0 to 1 is a valid rule for labeling active peptides for this data table. This rule forms a boundary between active and inactive peptides for this data table. In larger data tables, rules may also form boundaries between active peptides or between inactive peptides because different features may be relevant for the activity for different sets of peptides.

**Table 5:** Data table consists of six selected sequences with two features

Sequence	Sum of Sum of FAUJ880111	Sum of Sum of FAUJ880112	Antibacterial Activity
FFPVIGRILNGIL	1	0	Active
KFHEKHHSHRGY	3	1	Active
GNNRPVYIPQPRPPHPRL	3	0	Active
QDVDHVFLRF	1	2	Inactive
QQDYTGWMDF	0	1	Inactive
QLTFTSSWG	0	0	Inactive

### Correlated AAindex1 property removal

The AAindex1 has 544 properties with one value for each of the twenty naturally occurring amino acids [63]. A database of all properties is available in the R package ‘seqinr’ [73]. We constructed an autocorrelation matrix of these properties to provide pairwise correlation comparisons for all 544 properties. We filtered properties using an absolute correlation value cutoff. We randomized which property to keep by randomizing the order in which the properties were compared.

### Performance descriptions

In binary classification there are two different descriptions of performance based on the two possible error types, false positives and false negatives. Sensitivity refers to the likelihood of correctly predicting a positive result, while specificity refers to the likelihood of correctly predicting a negative result. Sensitivity deals with avoiding false positives, while specificity deals with avoiding false negatives. Selectivity, which can be directly derived from specificity, is the likelihood of incorrectly predicting a negative result, a false negative. Further details about performance measures are included in Additional file 1.

## Additional file

Additional file 1: Feature Generation and Performance Measure Methods (DOCX 30 kb)

## Abbreviations

AAindex1: Amino acid index 1
AMP: Antimicrobial peptide
ANN: Artificial neural network
APD2: Antimicrobial peptide database 2
CAMP: Collections of antimicrobial peptides
CLN: Condition limit number
DA: Discriminant analysis
EFC + 307-FCBF: Evolutionary feature construction and fast correlation-based filter selection with 307 features
EFC: Evolutionary feature construction
EFC-FBCF: Evolutionary feature construction without fast correlation-based filter selection
FBCF: Fast correlation-based filter selection
FDR: False discovery rate
FN: False negative
FP: False positive
HMM: Hidden Markov model
iAMP-2 L: Antimicrobial peptide prediction two-level
IRIM: Interesting rule induction method
LR: Logistic regression
MCC: Matthew’s correlation coefficient
MLEM2: Modified Learning from Experience Module 2
QM: Quantitative matrix
SVM: Support vector machine
TN: True negative
TP: True positive
