# Supplementary material for: Antimicrobial peptide similarity and classification through rough set theory using physicochemical boundaries
Source: BMC Bioinformatics. 2018 Dec 6;19:469. doi: 10.1186/s12859-018-2514-6 (PMC6282327; doi:10.1186/s12859-018-2514-6)
Supplement: Supplementary file 1 — Feature Generation and Performance Measure Methods (DOCX 30 kb) [file 12859_2018_2514_MOESM1_ESM.docx]

Additional file 1

# Antimicrobial Peptide Similarity and Classification through Rough Set Theory Using Physicochemical Boundaries

Authors:

Kyle Boone

1530 W 15th Street

Learned Hall, Room 5109

Lawrence, KS 66045

Bioengineering Program, University of Kansas

Institute of Bioengineering Research, University of Kansas

kyle.boone@ku.edu

Kyle Camarda

1530 West 15th Street

Learned Hall, Room 4154

Lawrence, KS 66045

Chemical and Petroleum Engineering Department, University of Kansas

camarda@ku.edu

Paulette Spencer

1530 West 15th Street

Learned Hall, Room 3111

Lawrence, KS 66045

Mechanical Engineering Department, University of Kansas

Bioengineering Program, University of Kansas

Institute of Bioengineering Research, University of Kansas

pspencer@ku.edu

Candan Tamerler (corresponding author)

1530 W 15th St

Learned Hall, Room 3135A

Lawrence, KS 66045

Mechanical Engineering Department, University of Kansas

Bioengineering Program, University of Kansas

Institute of Bioengineering Research, University of Kansas

[ctamerler@ku.edu](mailto:ctamerler@ku.edu)

**Feature Generation**

Datasets for this classification system are lists of peptide sequences. Each peptide sequence is a list of letters representing amino acids in a polypeptide chain. The lengths of peptide sequences vary in the dataset. Features for this classification system are single number summaries of the entire peptide sequence. See Table S1.

**Table S1: Description of summary functions to generate chemical property features as input for the MLEM2 method.**

| *Summary Function* | *Description* |
| --- | --- |
| Sum | $\sum a_{i}$, where *a_i_* is the amino acid chemical property at position *i* from 1 to *n*, the length of the peptide sequence |
| Mean | $\sum\frac{a_{i}}{n}$, where *a_i_* is the amino acid chemical property at position *i* from 1 to *n* |
| Window 3 | $max({\sum_{i}^{i+3} a}_{i}$), where *a_i_* is the amino acid chemical property at position *i* from 1 to *n-3* |

**Performance Measures**

When measuring the performance of a prediction, there are four possible prediction outcomes for each sequence predicted: true positive (TP), false positive (FP), true negative (TN) and false negative (FN). True refers to correct classification while false refers to a misclassification. Positive refers to a classification of antibacterial and negative refers to a classification of non-antibacterial. We used multiple performance measures used to understand how well a classifier can predict positive or negative cases. The first is sensitivity, which is the frequency of predicting antibacterial sequences as antibacterial.

$$Sensitivity=\frac{TP}{TP+FN}$$

The second type is specificity, which is the frequency of predicting non-antibacterial sequences as non-antibacterial. False discovery rate is its complement, the frequency of predicting non-antibacterial sequences as antibacterial.

$$Specificity= \frac{TN}{TN+FP}=1-False Discovery Rate$$

Lastly, we used a performance measure that combines elements of sensitivity and specificity into a single measure called the Matthew’s Correlation Coefficient (MCC). This measure is derived from the Pearson correlation coefficient for predictions when classifications are binary.^1^ Because it is a special case of Pearson’s correlation, the maximum value is 1 when the prediction is perfect, 0 when random and -1 when all predictions are false.

$MCC= \frac{TP*TN-FP*FN}{\sqrt{(TP+FP)(TP+FN)(TN+FP)(TN+FN)}}$

**References**

[1] P. Baldi, S. Brunak, Y. Chauvin, C.A.F. Andersen, H. Nielsen, Assessing the accuracy of prediction algorithms for classification: an overview, Bioinformatics 16(5) (2000) 412-424.
